# Non-penetrant Xq26.3 duplication involving the invariant TAD border: clinical evidence for the *VGLL1* region as the *GPR101* pituitary enhancer of X-linked acrogigantism

**DOI:** 10.1007/s11102-025-01559-4

**Published:** 2025-07-20

**Authors:** Cathie Hilditch, Samuel Curtis, Samuel Cotton, Shannon LeBlanc, Sunita De Sousa

**Affiliations:** 1 Paediatric and Reproductive Genetics Unit, Women’s and Children’s Health Network, North Adelaide, South Australia; 2 Adelaide Medical School, The University of Adelaide, Adelaide, South Australia; 3 Department of Genetics and Molecular Pathology, SA Pathology, Adelaide, South Australia Australia; 4 Endocrine and Metabolic Unit, Royal Adelaide Hospital, Adelaide, South Australia; 5 South Australian Adult Genetics Unit, Royal Adelaide Hospital, Adelaide, South Australia

**Keywords:** *GPR101*, X-linked acrogigantism, Pituitary adenoma, Topologically associating domains, TADopathy, *VGLL1*

## Abstract

**Introduction:**

X-linked acrogigantism (X-LAG; OMIM: 300942) is a rare X-linked dominant, fully penetrant form of infancy-onset pituitary gigantism caused by Xq26.3 tandem duplications involving the *GPR101* gene. All previously reported X-LAG-associated duplications disrupt the integrity of the resident topologically associating domain (TAD). This creates a neo-TAD, permitting ectopic chromatin interactions between *GPR101* and centromeric pituitary enhancers postulated to lie between *RBMX* and *VGLL1*, and culminating in pituitary *GPR101* misexpression and growth hormone excess. Conversely, none of the few previously reported cases of Xq26.3 duplications in unaffected individuals include the tissue-invariant TAD border that shields *GPR101* from its centromeric enhancers. Preservation of this boundary has thus been considered synonymous with non-penetrance of X-LAG.

**Methods:**

We examined a series of four family members from the same kindred with an incidentally detected *GPR101*-containing Xq26.3 duplication involving the invariant TAD border.

**Results:**

Chromosome microarray demonstrated an interstitial chromosome Xq26.3 duplication: arr[GRCh37] Xq26.3(135,954,223 − 136,224,319)x2, including *GPR101*, the TAD invariant border and *RBMX*, but not *VGLL1*. None of the relatives with the Xq26.3 duplication exhibited evidence of growth hormone excess, making this the first unaffected family with a *GPR101*-containing Xq26.3 duplication involving the invariant TAD border. The predicted neo-TAD in this kindred excludes the *VGLL1* region, which is present in all previously described X-LAG patients and absent in all previously described unaffected individuals with Xq26.3 duplications.

**Conclusion:**

Our clinical findings suggest that TAD border involvement is not sufficient for X-LAG to develop, and implicates the *VGLL1* region as likely the sole pituitary enhancer responsible for *GPR101* misexpression and the X-LAG phenotype. Pending corroborative studies, this new insight into X-LAG pathogenesis may guide interpretation of future Xq26.3 duplications and counselling of families in whom such duplications are found.

## Introduction

X-linked acrogigantism (X-LAG; OMIM #300942) is an X-linked dominant, fully penetrant form of pituitary gigantism that begins soon after birth [1, 2]. It is characterised by growth hormone (GH) hypersecretion – commonly with prolactin cosecretion – by pituitary adenomas or less frequently pituitary hyperplasia [1]. The underlying genetic defect is germline or somatic mosaic tandem Xq26.3 microduplications, which vary in precise positioning but consistently involve the *GPR101* gene (OMIM *300393) encoding an orphan G-protein-coupled receptor [1, 3, 4]. X-LAG is classified as a TADopathy [3], with disruption of the topologically associating domains (TADs) that normally guide chromatin folding in the nucleus and segregate genomic regions, thereby facilitating highly specific enhancer-promoter physical DNA interactions [3, 5]. Under normal conditions, *GPR101* is insulated from nearby genes and regulatory sequences by a centromeric tissue-invariant TAD border [3]. In X-LAG, the causative duplications create a neo-TAD, positioning enhancer regions adjacent to *GPR101* without separation by the TAD border, leading to ectopic enhancer-promoter interactions and pituitary *GPR101* misexpression [3]. This manifests as marked GPR101 upregulation in X-LAG pituitary lesions [1], and GPR101 in turn directly stimulates GH and prolactin hypersecretion [6].

Three clinically unaffected kindreds with *GPR101*-containing duplications were recently reported in 2024 by Daly et al. [7]. The invariant TAD border was not included in these ‘neutral’ duplications, supporting the importance of TAD boundary rearrangements in producing the ectopic enhancer-*GPR101* promoter interactions that drive GH excess [7]. By comparing X-LAG and clinically unaffected Xq26.3 duplication cases, and examining physical DNA interactions through 3D genome technologies, Daly et al. postulated that the pituitary enhancers interacting with *GPR101* in the neo-TAD lie between *RBMX* and *VGLL1* [7].

Herein we report a clinically unaffected kindred with an Xq26.3 duplication involving *GPR101*, the invariant TAD border and *RBMX*, but not *VGLL1*, suggesting that the *VGLL1* region (or part thereof) is the essential pituitary enhancer for *GPR101* misexpression and the X-LAG phenotype.

## Case series 

### IV-1

**Fig. 1:**
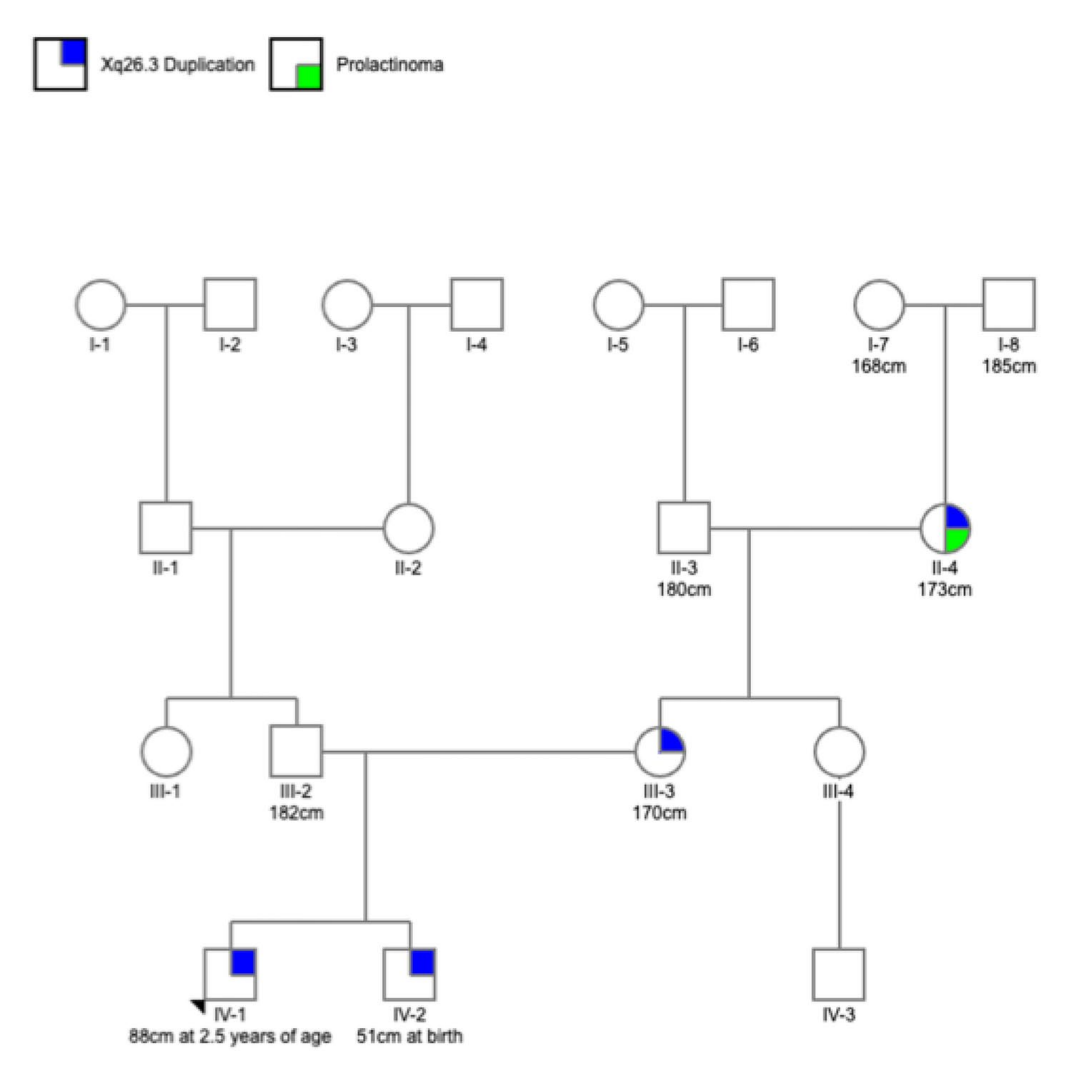
Family pedigree including relevant heights. Adult height is given unless otherwise specified. None of the four individuals with the Xq26.3 duplication had evidence of overgrowth. II-4 had a young-onset prolactinoma without evidence of growth hormone excess.

The proband (IV-1) is a boy who was assessed in a local clinical genetics service at 2 years and 5 months of age due to mild gross motor and expressive speech delay. On examination, his weight was 13.7 kg (50-75th centile), height 88 cm (25-50th centile) and head circumference 49 cm (50th centile). There was no facial dysmorphism or abnormalities of the hands or feet to suggest overgrowth. Cardiorespiratory, abdominal and neurological examination was unremarkable. Chromosome microarray performed in investigation of his developmental delay showed a male profile with an unexpected 270 kb interstitial chromosome Xq26.3 duplication: arr[GRCh37] Xq26.3(135,954,223 − 136,224,319)x2, including OMIM-morbid genes OMIM-morbid genes *GPR1010* (OMIM*300393) and *RBMX* (OMIM 300199) but, notably, not *VGLL1* (OMIM *300583). His pedigree is shown in Fig. 1 and relevant family members are discussed below. He was 3 years and 4 months of age at last review – height remained at the 25-50th centile and his mild gross motor delay continued, although his expressive speech delay had resolved.

### III-3

Parental testing identified that IV-1’s mother, III-3, also carried the Xq26.3 duplication. III-3 was of average adult height (170 cm), with no acromegalic features or clinical history to suggest pituitary dysfunction at any stage.

### II-4

Further segregation testing showed that IV-1’s maternal grandmother, II-4, carried the Xq26.3 duplication. Whilst there was no evidence of an overgrowth phenotype (adult height 173 cm), II-4 had been diagnosed with a prolactinoma at age 25 years after presenting with infertility, galactorrhoea and visual field abnormalities. She was transiently treated with bromocriptine; recent biochemical screening off dopamine agonist therapy showed normal serum prolactin and IGF-1 levels at age 61 years.

### IV-2

Segregation testing also showed that IV-1’s younger brother, IV-2, carried the Xq26.3 duplication. IV-2 was of average length at birth (51 cm, 50th centile). He was 9 months of age at least review with normal growth and development.

## Discussion

**Fig. 2:**
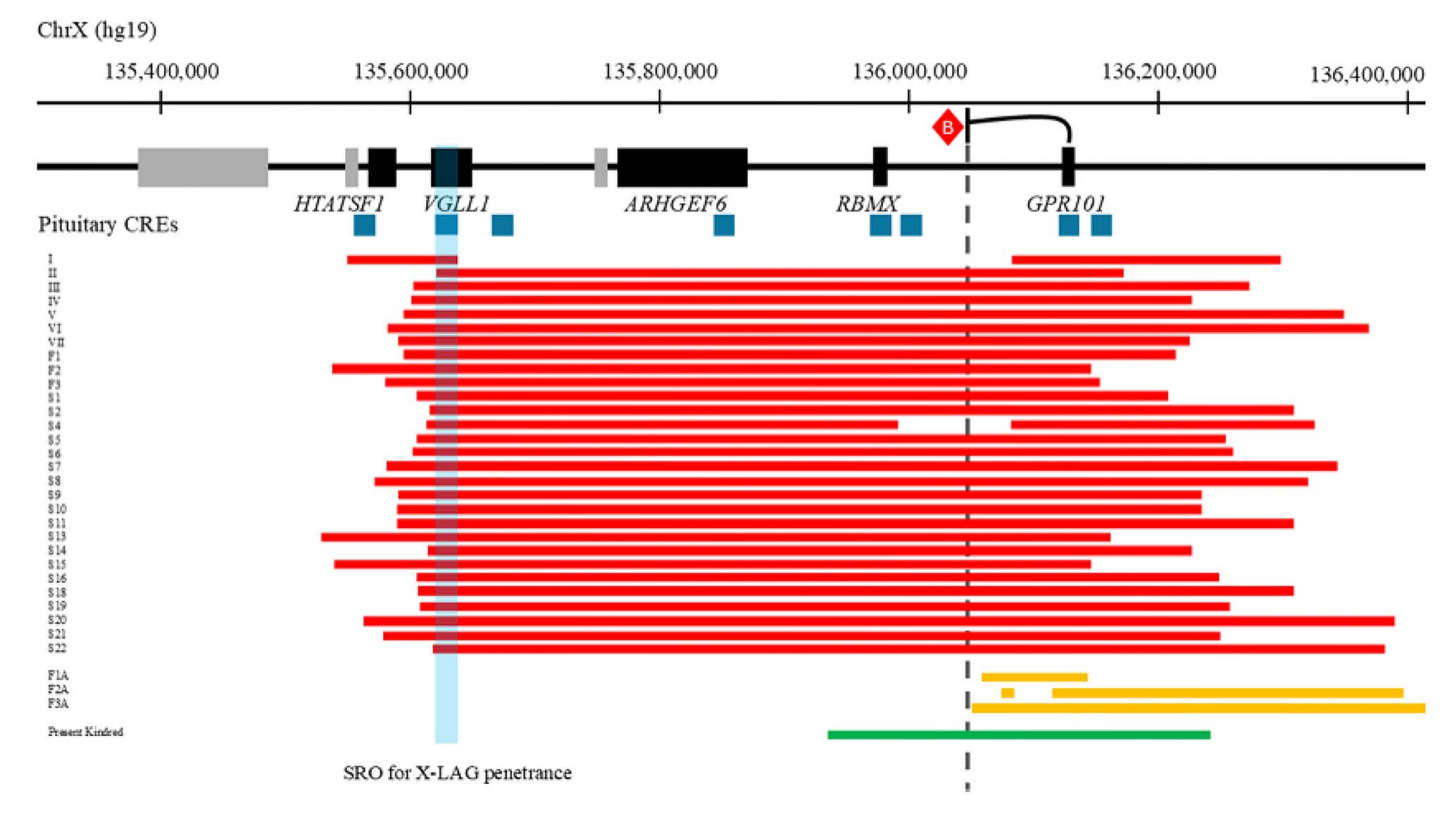
Positions of Xq26.3 duplications reported to date. Each horizontal bar represents a reported duplication. Kindred naming is in accordance with prior conventions [3]. Penetrant duplications are shown in red. Non-penetrant duplications are either yellow, from [7], or green, representing the current kindred. The blue column indicates the only region to be present in all penetrant duplications and absent in all non-penetrant duplications to date; this contains only one of the previously proposed candidate pituitary CREs – the *VGLL1* region. Abbreviations: CRE, cis regulatory elements SRO, smallest region of overlap.

**Fig. 3:**
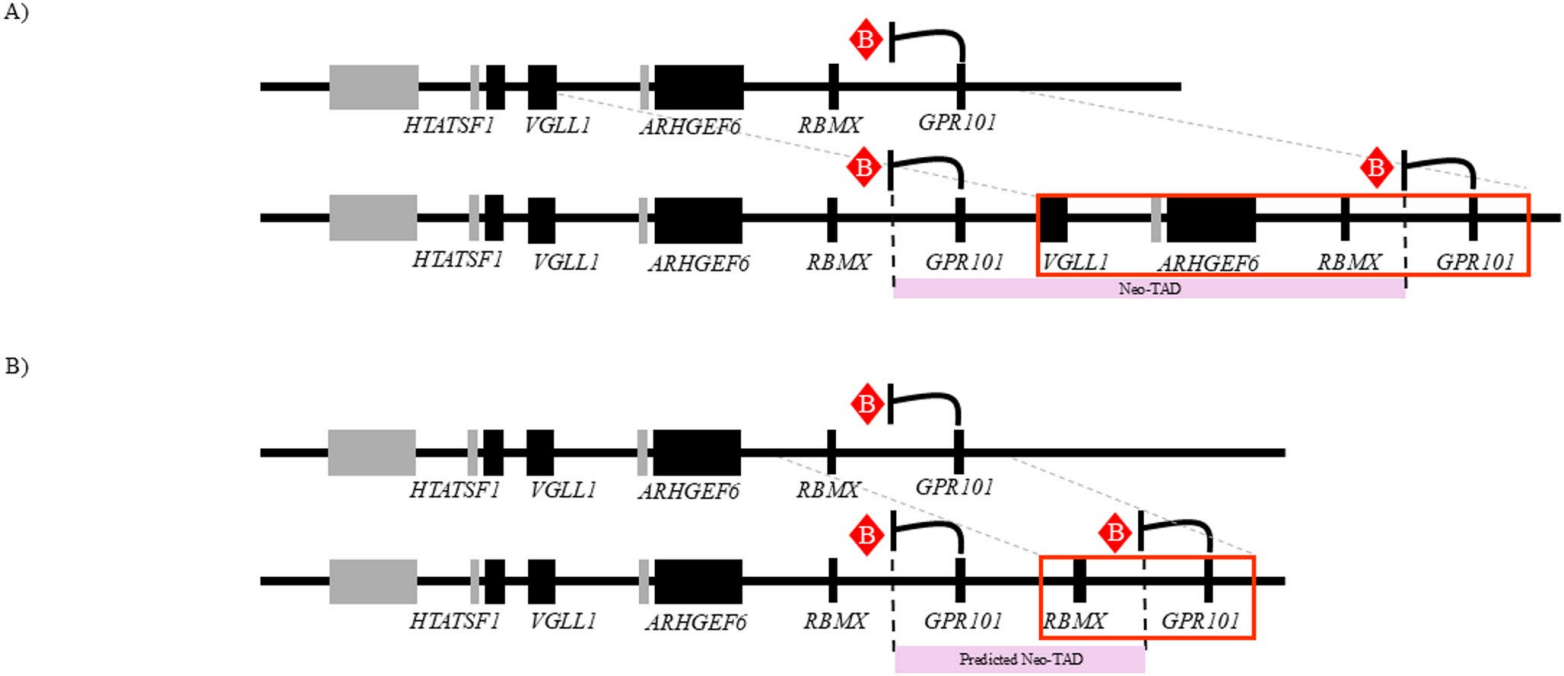
Neo-TAD configurations in penetrant X-LAG (**A**) versus the present unaffected kindred (**B**). Schematic diagrams of the expected neo-TADs bound by the native and duplicated invariant TAD borders are shown. The predicted neo-TAD in the present unaffected kindred is smaller and does not contain the *VGLL1* enhancer regions of interest from existing literature. Abbreviations: TAD, topologically associating domain; X-LAG, X-linked acrogigantism

The critical components of X-LAG penetrance have been progressively elucidated through informative clinical cases, such as the family presented herein, in addition to sophisticated investigations of chromatin structure [7]. As illustrated in Fig. 2, there is a telomeric component containing *GPR101* in all X-LAG cases, but also unaffected cases, and a centromeric component consistently containing the *VGLL1* region in all X-LAG cases and no unaffected cases [3, 7]. *VGLL1* is the only candidate pituitary enhancer region present in all X-LAG cases and no unaffected cases – the other compelling candidate is *RBMX* which is present in all but one X-LAG cases and absent in all previously reported unaffected cases [3, 7]. Involvement of the centromeric tissue-invariant TAD border is present in most but not all X-LAG cases and absent in all previously reported unaffected cases [3, 7]. Thus, our unaffected family’s duplication containing *RBMX* and the invariant TAD border but not the *VGLL1* region, as depicted in Fig. 3, uniquely suggests that *GPR101* interactions with the *VGLL1* region are vital for *GPR101* misexpression and X-LAG penetrance.

Studying clinically unaffected Xq26.3 duplication cases is critical to the interpretation of future cases, particularly in the preimplantation/IVF and prenatal settings where phenotype of the tested individual is unknown. Between the present report and the 2024 report by Daly et al. [7], it appears that lack of either the *VGLL1* region or the TAD border may indicate a non-penetrant duplication. Ideally, chromatin conformation capture studies would be applied in such cases to exclude the ectopic enhancer-promoter patterns characteristic of X-LAG [7]. However, these studies are labour and time intensive and require special expertise. In silico models of structural variants and TAD-related effects may emerge as a more accessible tool [7].

Better prediction of non-penetrant duplications may also circumvent unnecessary pituitary tumour surveillance in adults which may otherwise be pursued given limited familiarity with this very rare condition (~ 50 X-LAG cases reported to date [2]). Interestingly, the grandmother in the present kindred had a young-onset, presumably large prolactinoma as may be seen in familial pituitary tumour conditions [8]. This raises the intriguing possibility of a *GPR101* gene dosage effect contributing to pituitary tumorigenesis separate to the misexpression mechanism of X-LAG. However, without a tumour specimen to interrogate, and in the absence of other such cases, this prolactinoma is best considered a phenocopy unrelated to *GPR101*.

A limitation of this case study is that the duplication was assumed rather than proven to be tandem. If the observed duplication was inserted somewhere in the genome other than Xq26.3, then this might be an explanation for non-penetrance, although microduplications are typically tandem [9]. We were also unable to perform chromatin conformation capture studies to prove the assumed lack of ectopic enhancer adoption in the neo-TAD. Whilst not available in our institution, the next steps to evaluate the hypothesis proposed herein would ideally involve chromatin conformation capture and long-read sequencing to characterise non-penetrant duplications at maximal precision.

In conclusion, we report the first unaffected kindred with a *GPR101*-containing Xq26.3 duplication involving the invariant TAD border. This duplication also contains *RBMX* but not *VGLL1*, suggesting that *GPR101* interactions with the *VGLL1* region may be vital for *GPR101* misexpression and X-LAG penetrance. This novel finding should guide further research into the pathogenesis of X-LAG and, more broadly, pituitary tumorigenesis. Pending further clinical and experimental studies through international collaborative efforts, this hypothesis may inform the clinical management of future Xq26.3 duplication cases.

## Data Availability

No datasets were generated or analysed during the current study.
